# Attitudes about withholding or withdrawing life-prolonging treatment, euthanasia, assisted suicide, and physician assisted suicide: a cross-sectional survey among the general public in Croatia

**DOI:** 10.1186/s12910-022-00751-6

**Published:** 2022-02-17

**Authors:** Ana Borovecki, Marko Curkovic, Krunoslav Nikodem, Stjepan Oreskovic, Milivoj Novak, Filip Rubic, Jurica Vukovic, Diana Spoljar, Bert Gordijn, Chris Gastmans

**Affiliations:** 1 School of Medicine, Center for Palliative Medicine, Medical Ethics and Communication Skills, University of Zagreb, Salata 2, 10000 Zagreb, Croatia; 2 School of Medicine, University Psychiatric Hospital Vrapče, University of Zagreb, Zagreb, Croatia; 3 Faculty of Humanities and Social Sciences, Department of Sociology, University of Zagreb, Zagreb, Croatia; 4 School of Medicine, Department of Medical Sociology, University of Zagreb, Zagreb, Croatia; 5 School of Medicine, University Hospital Centre Zagreb, University of Zagreb, Zagreb, Croatia; 6 School of Medicine, University Hospital Dubrava, University of Zagreb, Zagreb, Croatia; 7 Institute of Ethics, School of Theology, Philosophy, and Music, Dublin City University, Dublin, Ireland; 8 Centre for Biomedical Ethics and Law, Faculty of Medicine, KU Leuven, Leuven, Belgium

**Keywords:** End-of-life decision-making, Euthanasia, Assisted suicide, Physician assisted suicide, Withholding, Withdrawing, Attitudes, Croatia

## Abstract

**Background:**

There has been no in-depth research of public attitudes on withholding or withdrawing life-prolonging treatment, euthanasia, assisted suicide and physician assisted suicide in Croatia. The aim of this study was to examine these attitudes and their correlation with sociodemographic characteristics, religion, political orientation, tolerance of personal choice, trust in physicians, health status, experiences with death and caring for the seriously ill, and attitudes towards death and dying.

**Methods:**

A cross-sectional study was conducted on a three-stage random sample of adult citizens of the Republic of Croatia, stratified by regions, counties, and locations within those counties (N = 1203). In addition to descriptive statistics, ANOVA and Chi-square tests were used to determine differences, and factor analysis (component model, varimax rotation and GK dimensionality reduction criterion), correlation analysis (Bivariate correlation, Pearson’s coefficient) and multiple regression analysis for data analysis.

**Results:**

38.1% of the respondents agree with granting the wishes of dying people experiencing extreme and unbearable suffering, and withholding life-prolonging treatment, and 37.8% agree with respecting the wishes of such people, and withdrawing life-prolonging treatment. 77% of respondents think that withholding and withdrawing procedures should be regulated by law because of the fear of abuse. Opinions about the practice and regulation of euthanasia are divided. Those who are younger and middle-aged, with higher levels of education, living in big cities, and who have a more liberal worldview are more open to euthanasia. Assisted suicide is not considered to be an acceptable practice, with only 18.6% of respondents agreeing with it. However, 40.1% think that physician assisted suicide should be legalised. 51.6% would support the dying person’s autonomous decisions regarding end-of-life procedures.

**Conclusions:**

The study found low levels of acceptance of withholding or withdrawing life-prolonging treatment, euthanasia, assisted suicide and physician assisted suicide in Croatia. In addition, it found evidence that age, level of education, political orientation, and place of residence have an impact on people’s views on euthanasia. There is a need for further research into attitudes on different end-of-life practices in Croatia.

## Background

The six groups of end-of-life practices usually distinguished in the literature are: intensified alleviation of symptoms with potentially life-shortening effects, withholding/withdrawing life-prolonging treatment, continuous deep sedation until death, euthanasia defined as administration of a lethal injection by a physician at the explicit request of the patient, physician-assisted suicide (suicide by a patient facilitated by means (such as a drug prescription) or by information (such as an indication of a lethal dosage), provided by a physician aware of the patient's intent), and ending of life without explicit patient consent [1–4]. Some of these practices are more controversial than others. While palliative care practices enjoy widespread acceptance across Europe, certain end-of-life practices, such as euthanasia and physician-assisted suicide, have been legalised in only a few European countries and have triggered ethical debates all over the world [5–7].

Research shows an increasing incidence of withholding (WTH) or withdrawal (WTD) of life-prolonging treatments, as well as procedures aimed at alleviating pain or other symptoms with life-shortening side effects [5, 6, 8–10]. The results of empirical research over the last decade, both in Europe and in the US, also show a general trend of increasing acceptance of euthanasia and physician-assisted suicide. Studies conducted by Cohen et al. and those based on the European Values Study project, show that in the last 20 years approval of euthanasia by the general population in Europe has increased from 25 to 33% [11, 12]. However, the trajectories for specific countries may vary. Acceptance levels of euthanasia and physician assisted suicide peaked in most countries, and in some countries a moderate decline was reported [6, 7, 11, 12].

Studies single out numerous possible factors that influence public attitudes towards end-of-life practices, such as individual sociodemographic characteristics, health status, political orientation, the legal or healthcare situation of a country, and trust in institutions [5, 6, 11, 12]. These and many other possible factors interrelate in complex ways in different populations and contexts, which makes them difficult to generalize.

Comparatively speaking, Croatia today is the country with the lowest institutional trust and one of the lowest levels of societal trust in Europe. According to the results of the European Values Study for 2017, only 7.2% of Croatian citizens trust the parliament, 9.6% trust the government, and 13.6% trust the judiciary. Confidence in the health care system is still high. 41.6% of citizens have confidence in the health care system. Only 13.6% of Croatian citizens trust other people [13]. Although Croatian society is still mostly traditional in the last twenty years, there have been changes in terms of secularization and liberalization. Thus, the number of citizens who believe that abortion and euthanasia can never be justified decreased from 55 to 47%, which is still a high percentage compared to developed European countries, where about 15% of citizens think the same. Also, the negative attitude towards homosexuality decreased, from 75% in 1999 to 62% in 2017. In parallel, in developed European countries it is about 11% [14].

In a religious sense, Croatia is a country with a majority of citizens who consider themselves to be Catholics. According to the census of 2011, 91.37% of the population declared themselves to be members of different Christian denominations (of which 86.28% were Catholic). 1.44% of the population declared themselves to be Muslim and 0.01% Jewish [14].

The ethical dilemmas most frequently experienced by Croatian physicians and nurses in end-of-life practices have to do with the uncertain or impaired decision-making capacity of patients and the withdrawing or withholding of life-prolonging treatment at the end of life [15]. The practices of withholding or withdrawing life-prolonging treatments are not regulated by law in Croatia. Nevertheless, they are present in everyday work of physicians in Croatia. It is usually the medical team (mainly physicians) that makes decisions regarding withholding or withdrawing life-prolonging treatments. In the Code of Medical Ethics and Deontology of the Croatian Medical Chamber and the Croatian Medical Association, in a short paragraph on the dying patient, the following recommendation is found: “Continuing intensive treatment of a patient in an irreversible final condition is not medically necessarily well-founded, and excludes the right of the dying patient to a dignified death.”

In the Republic of Croatia, euthanasia and assisted suicide are illegal. In the legal provisions there is no mention of physician-assisted suicide, just a general description of assisted suicide is given. Euthanasia and assisted suicide are prescribed as criminal offences in the Criminal Code, with a punishment of up to three years in prison. However, some legal scholars are not happy with the current description of assisted suicide as a criminal act where one leads another to suicide or helps them to commit suicide for selfish motives (e.g. to gain inheritance or any other personal gain). In their interpretation, if the motives are not selfish but assisted suicide is committed out of compassion (e.g. helping someone to commit suicide because of their unbearable suffering without any personal gain) then the person who participated in it will probably not be prosecuted. This prompts the legal question: Is assisted suicide as end-of-life practice legalised in Croatia [16]? As for anticipatory decision-making (e.g., advance directives, do-not-resuscitate orders), *de iure* provisions for this are envisaged in family law, but de facto in actual legal implementation there are no additional legal provisions that would enable the implementation of anticipatory decision-making by patients in Croatia [17]. Previous research in Croatia regarding attitudes towards euthanasia put Croatia among the countries with a low level of public acceptance of this practice [7, 11, 12].

To date no in-depth research has been done on public attitudes towards end-of-life practices in the Republic of Croatia. Thus, the first aim of this study was to examine public attitudes towards withholding or withdrawing life-prolonging treatment, and euthanasia, assisted suicide and physician-assisted suicide. Further, the study also analysed the correlations between these attitudes and sociodemographic characteristics, religion, political orientation, tolerance of personal choice, trust in physicians, health status, experiences with death and caring for seriously ill patients, and attitudes towards death and dying.

## Methods

This study is part of a research project entitled: ‘Values and Decisions at the End of Life’, (VAL-DE-END). The aim of this project is to conduct a comprehensive analysis of end-of-life issues in ICUs in tertiary level healthcare institutions in the Republic of Croatia. Whilst the project’s focus is on ICUs, the project also involves a research strand directed at the Croatian population. This research was done as an omnibus survey of the general Croatian population, aimed at covering several topics (issues of attitudes towards death and dying, end-of-life decision-making, end-of-life practices, trust in physicians and healthcare, attitudes to other bioethical issues.). We have already published one paper based on a survey of the general Croatian population on what constitutes a good death [18]. In the current paper we present data from the same survey on the Croatian public’s attitudes towards practices at the end of life. The data that we present in this paper do not include 13 items in our analysis. Five case- vignettes that dealt with cases of patients where end-of-life decision-making was required were excluded for methodological reasons. We plan to analyse and to publish this data in the future. We also did not include in the analysis in this paper for thematic reasons eight items dealing with general attitudes towards other bioethical issues (genetics, use of technology, etc.).

### Design

We conducted a descriptive cross-sectional survey study.

### Sample

The research was conducted on a three-stage random sample, stratified by regions, counties and locations within those counties. The sample (N = 1203) of adult citizens of the Republic of Croatia was constructed in accordance with the 2011 census. The stated number of respondents at the overall level allows inference to the target population, with a maximum sample error of ± 2.8%. The response rate was 30%.

By including weights, the sample became nationally representative in terms of sex, age, education and regional representation. Sampling methods used in the research included stratified random sampling. The required number of respondents in each county was predefined according to the proportional share of the population. Settlements (cities, towns, villages) were randomly selected, taking into account the rural–urban distribution in each county. The place of residence in each settlement was randomly selected by the random walk method, in which randomly selected surveyors entered every third household on their right side. In each household, the last birthday method was used as a selection criterion. After initial contact with a household by the surveyors and the detection of potential respondents, when they agreed to participate in the survey, the surveyors explained the background of the study and the goals of the study to the participants. Participation in the study was voluntary, and the participants were given information about data protection and assurance of data anonymity and confidentiality. The questionnaire was administered by trained surveyors. The survey was conducted in November and December 2019 [18].

### Instrument

The questionnaire was developed as a part of the VAL-DE-END project, for the purpose of examining the attitudes of the population and aimed to cover several themes (an omnibus questionnaire covering issues relating to attitudes towards death and dying, end-of-life decision-making, end-of-life practices, trust in physicians and healthcare, and attitudes to other bioethical issues.) The questionnaire was developed over several consecutive phases. Firstly, an extensive literature search was performed to identify and analyse existing questionnaires used for similar purposes. Items informed by the literature review were assessed by experts in survey methodology, and experts from the respective fields of study. All items that were originally available in English were translated twice from English to Croatian by independent translators, followed by translations back to English by two additional translators. The questionnaire was pre-tested on a small, convenience sample of the target population (n = 50) [18].

The final questionnaire has 90 items. However, for the purpose of the current paper we have included 77 items from the questionnaire for our analysis. In this analysis we focused mainly on attitudes about withholding or withdrawing life-prolonging treatment, euthanasia, physician assisted suicide, and assisted suicide.

In order to provide a clear overview of the results we decided to divide the 77 included items into four groups. The first group is based on the items of the European Values Study project [19]. It consists of 10 socio-demographic items which together aim to measure gender, age, marital status, education level, the education level of parents, household income, profession, number of descendants, and the size of place of residence. We also adapted 12 items from the European Values Study to measure religiosity and religious beliefs, political orientation, and tolerance of personal choice.

As trust is an important factor in the physician–patient relationship and end-of-life practices, a group of 18 items attempt to measure trust in institutions and trust in physicians and healthcare, and the health status of the respondents. These items stem from the European Values Study [19] project and research by Dugan et al. [20] and Hall et al. [21].

The third group consists of 24 items dealing with attitudes about and experiences with death and dying, informed by the study by Nikodem [22] and the study by Yun et al. [23].

The fourth group is made up of 7 items in total, and deals with attitudes to end-of-life practices that can fall under the following categories, according to van der Heide et al. [2]: withholding or withdrawing life-prolonging treatment with or without the patient’s consent, euthanasia, and physician-assisted suicide. This part of the questionnaire included items measuring general attitudes about the necessity for legal regulation of end-of–life practices, individuals’ right for self-determination about end-of-life issues, and the physicians’ corresponding obligations. The next part of the questionnaire included 6 items which aimed to measure the level of agreement with certain end-of-life practices. These, in total 13 slightly modified items, stem from the research and instruments constructed for previous studies [5, 24–26].

Instead of using the terms “euthanasia”, “assisted suicide” and “physician assisted suicide” we described practices, since these end-of life practices are controversial, and if explicitly mentioned may immediately galvanise the respondents’ replies. We used the following descriptions for euthanasia practices: „ “a procedure in which a person is directly killed by a physician”, “a physician giving a substance which will cause the death of people who are dying and who are experiencing extreme suffering” and “to painlessly end the life of a patient who is suffering from an incurable illness”. For assisted suicide we used the following description: “enabling people who are dying and who are experiencing extreme suffering to end their own lives”. For physician assisted suicide we used the following description: “a physician helping a patient who is suffering from an incurable illness and is living with severe pain to end their own life”.

Replying to the questionnaire was voluntary and was not rewarded. The anonymity of the participants’ data was ensured.

### Data analysis

Data were processed in IBM SPSS Statistics 26. In addition to descriptive statistics, we used ANOVA. We started from the assumption that attitudes about end-of-life practices are not one-sided, one-dimensional, but that they are multidimensional. Thus we assumed that in the background of these attitudes there were latent dimensions (factors, patterns of thinking about end-of-life practices). We conducted a factor analysis (component model, varimax rotation and GK dimensionality reduction criterion) to determine the structure of these latent dimensions (factors, patterns of thinking). Factor analysis and the Chi-square were also used to test differences in respondents’ answers in relation to the basic sociodemographic characteristics of the respondents Multiple regression analysis and correlation analysis (bivariate correlation, Pearson’s coefficient) were used to find predictors of and links with factors (patterns of thinking about end-of-life practices).

## Results

### Sociodemographic characteristics of the respondents

1203 respondents participated in the study. The average age of the respondents was 48.21 years. More than half of the respondents were females. 43% of the respondents were married, and slightly fewer than half of the respondents had either one child or two children. More than half of the respondents had some form of secondary education (vocational school, high school). Around two thirds of the respondents were employed. 43.3% of the respondents lived in settlements with fewer than 2000 inhabitants. 35.3% of the respondents had incomes below the average income in the Republic of Croatia (Table 1).

**Table 1:** Sociodemographic characteristics of the respondents (N = 1203)

Sample characteristics	N	%
**Gender**		
Male	572	47.6
Female	631	52.4
**Marital status**		
Married	517	43
Not married	279	23.2
Divorced	145	12.1
Widowed	159	13.2
Extramarital union	77	6.4
**Number of children**		
Childless	389	32.2
One child	240	20
Two children	343	28.5
Three children	134	11.2
Four children	70	5.9
Five to seven children	17	1.4
**Education**		
Unfinished primary school	79	6.6
Primary school (8 years)	257	21.4
Secondary vocational (1–3 years)	239	19.9
Secondary vocational (4 years and longer)	318	26.4
High school	103	8.6
2–3 years of higher education	69	5.7
College	110	9.1
Master’s degree	23	1.9
PhD degree	4	0.3
**Employment**		
Employed	789	65.6
Unemployed	28	2.3
Retired	245	20.4
**Type of settlement**		
Less than 2000 inhabitants	521	43.3
Between 2–10,000 inhabitants	191	15.9
Between 10–50,000 inhabitants	152	12.6
Between 50–100,000 inhabitants	60	5
Between 100–500,000 inhabitants	121	10.1
With more than 500,000 inhabitants	158	13.1
Monthly income per household*		
Less than 5512.50 HRK	424	35.30
5512.50–11,025.00 HRK	372	30.90
11,026.00–22,050.00 HRK	224	18.70
22,051.00 HRK and more	27	2.30

*The average net monthly salary in November 2020 was HRK 6823, and the minimum salary for 2021 was HRK 3,400

### Respondents’ religious beliefs and practices, political orientation and tolerance

The majority of respondents said they believe in God and consider themselves to be religious. However, only 35.4% of the respondents attend religious ceremonies at least once a month or more. Although the majority of respondents did not classify themselves as either right or left-wing, or either conservative or liberal on the political spectrum, the majority of them approve of divorce but disapprove of abortion and homosexuality (Table 2).

**Table 2:** Religious beliefs and practices, political orientation, and tolerance of personal choice (N = 1203)

Sample characteristics	N	%
**Religious beliefs**		
Religious	781	64.9
Believe in god	914	76
Believe in life after death	616	51.2
Believe in heaven	585	48.7
Believe in hell	510	42.4
**Attendance of religious services**		
More than once a week	90	7.5
Once a week	208	17.3
Once a month	128	10.6
Only for religious holidays	229	19
Once a year	71	5.9
Less than once a year	174	14.5
Never	237	19.7
**Political orientation**		
Left	130	10.8
Right	230	19.1
Liberal	211	17.5
Conservative	123	10.2
**Tolerance**		
*Divorce*		
Justify	504	41.9
Do not justify	207	17.2
*Abortion*		
Justify	267	22.2
Do not justify	485	40.3
*Homosexuality*		
Justify	263	21.9
Do not justify	515	42.8

### Respondents’ heath status, level of trust in physicians and healthcare, and attitudes and experience of death and dying

The majority of respondents believe that they are in good or very good health and display high levels of trust in their GPs. They also have high levels of trust in physicians and the healthcare system in Croatia (Table 3). 44.7% of the general public believe that there is mutual trust and respect between physicians and patients in Croatia, and 47.5% believe that physicians discuss treatment options with their patients.

**Table 3:** Respondents’ health status, level of trust in physicians and healthcare, and attitudes and experience of death and dying (N = 1203)

Sample characteristics	N	%
**State of one’s health**		
Good and very good	628	52.2
Satisfactory	355	29.5
Very bad and bad	190	15.8
**Trust**		
Their GP	942	78.3
Physicians	789	65.6
Healthcare system	711	59.1
**Experiences with death**		
Death of a friend	628	52.2
Death of a father	606	50.4
Death of a mother	503	41.8
Death of brother/sister	304	25.3
Death of partner	263	21.8
Death of their own child	77	6.4
Death of a pet	703	58.5
**Characteristics of a good death**		
Absence of pain	661	54.9
Presence of loved ones	474	39.4
Lack of burden on loved ones	473	39.3
Having had a fulfilled meaningful life	453	37.7
Being in the presence of god	387	32.2
Being alert and aware	271	22.5
Having all treatment options	249	20.7
Solving unfinished business	174	14.5
Dying at home	161	13.4

The majority of respondents had experienced the death of a close person, father or mother, and only a small number had experienced the death of their own child. The death of a pet had been experienced by more than half of the respondents (Table 3). 42.2% of respondents had cared for a seriously ill person and 34.6% of respondents had cared for a terminally ill person. The absence of pain, and the presence of and lack of a burden on family and loved ones were selected as the most important characteristics of a good death by the respondents (Table 3).

47.1% of the respondents did not agree with the statement that everything stops with death. However, 69.1% of the respondents agreed with the statement that death is a natural, calm event after life. For 47.2% of the respondents agreed with the statement that death could only make sense when a person believes in God. 87.5% of the respondents found the meaning of life in friends and family, 66.6% in contributing to the life of the community, 64.1% in self-realization within their own possibilities, 56.7% in comfortable living, 37.8% in faith in God, and 22.2% in fulfilling their own desires. Only for 4.6% of respondents did life have no meaning.

### Attitudes to withholding/withdrawing life-prolonging treatment, euthanasia and assisted suicide

Table 4 shows the results at the level of distribution of the percentages of 13 statements that thematically cover end-of life decision-making.

**Table 4:** Distribution of respondents' answers to 13 statements in connection with end-of life decision-making*

Statements and answers	Number (%)
**Withholding or withdrawing life-prolonging treatment**	
1. Treatment procedures should not be initiated for people who are dying and who are experiencing extreme and unbearable suffering	
Agree	237 (19.7)
Don't know	303 (25.2)
Disagree	662 (55.1)
2. People who are dying and who are experiencing extreme and unbearable suffering should have their wish to die granted, and no treatment procedures should be initiated that could extend their life	
Agree	458 (38.1)
Don't know	310 (25.7)
Disagree	435 (36.2)
3. All treatment measures should be withdrawn from dying patients who are experiencing extreme and unbearable suffering	
Agree	174 (14.4)
Don't know	242 (20.1)
Disagree	787 (65.4)
4. People who are dying and who are experiencing extreme and unbearable suffering should be granted their wish to die, and all treatment measures that could prolong their life withdrawn	
Agree	455 (37.8)
Don't know	302 (25.1)
Disagree	445 (37.0)
5. Withholding treatment and "allowing" a patient to die should be regulated by law to avoid abuse	
Agree	927 (77.0)
Don't know	164 (13.7)
Disagree	112 (9.3)
**Euthanasia**	
6. Procedures in which a person is directly killed by a physician should be absolutely prohibited by law	
Agree	448 (37.2)
Don't know	296 (24.6)
Disagree	459 (38.2)
7. People who are dying and who are experiencing extreme suffering should be granted their wish to die by a physician giving them a substance which will cause their death	
Agree	361 (30.0)
Don't know	321 (26.7)
Disagree	521 (43.3)
8. Do you believe that physicians should be permitted by law to painlessly end the life of a patient who is suffering from an incurable illness, if the patient and the patient’s family ask for it?	
Agree	558 (46.4)
Don't know	205 (17.0)
Disagree	398 (33.1)
**Assisted suicide**	
9. People who are dying and who are experiencing extreme suffering should be granted their wish to die, and they should be enabled to end their own lives	
Agree	223 (18.6)
Don't know	290 (24.1)
Disagree	689 (57.3)
**10.Physician assisted suicide**	
Do you believe that physicians should be permitted by law to help a patient who is suffering from an incurable illness and is living with severe pain to end their own life, if the patient asks for it?	
Agree	483 (40.1)
Don't know	237 (19.7)
Disagree	430 (35.8)
**Patients’ rights and physicians’ obligations**	
11. A dying patient has the right to decide about the end of their own life	
Agree	620 (51.6)
Don't know	285 (23.7)
Disagree	297 (24.7)
12. No one, not even the individual in question, has the right to decide about the moment of their death	
Agree	426 (35.4)
Don't know	314 (26.1)
Disagree	463 (38.5)
13. Physicians are obliged to help a dying patient to realize their wish to die	
Agree	471 (39.1)
Don't know	343 (28.5)
Disagree	389 (32.3)

*We used the original 5-point scale for the items 1 to13 except for the items 8 and 10 where the 3-point scale was used. This may be the reason why we had missing answers on items 8 and 10 because larger range of possible answers gives a better selection of answers to respondents than 3 point-scale. In addition, items 8 and 10 are framed as questions and other items are framed as statements, which can also have an impact on missing answers. Some of the respondents may choose not to give answers to questions as they were framed in the items 8 and 10. Such framing of questions in a way forces them to be more explicit about legal regulation of physician assisted suicide and euthanasia

The original 5-point scale where 1 = strongly disagree and 5 = strongly agree were recoded to 3 degrees so that 1.2 = 1; 3 = 2, and 4.5 = 3. In table the positive responses are presented first, then indecisive and then negative responses.42 respondents (3.5%) did not give an answer to item number 8, which was then included in missing responses, while, 53 respondents did not give an answer to item number 10 (4.4%) which was also included in missing responses. There were no missing responses to the other items

Only 19.8% of the respondents think that treatment procedures should not be initiated (that treatment should be withheld) for people who are dying and who are experiencing extreme and unbearable suffering. Respectively, 14.4% agree with the statement that all treatment measures should be withdrawn from dying patients who are experiencing extreme and unbearable suffering.

A higher number of respondents (38.1%) think that people who are dying and who are experiencing extreme and unbearable suffering should have their wish to die granted, and no treatment procedures should be initiated that could extend their life. 37.8% of respondents agree with the statement that people who are dying and who are experiencing extreme and unbearable suffering should be granted their wish to die, and all treatment measures that could prolong their life withdrawn. The majority of respondents (77%) agreed with the statement that withholding treatment and "allowing" a patient to die should be regulated by law to avoid abuse.

Furthermore, 37.1% of the respondents agreed and 38.2% disagreed with the statement that procedures performed by a physician to directly kill a person should be absolutely prohibited by law. 46.4% of the respondents agreed with the statement that physicians should be allowed by law to painlessly end the life of a patient suffering from an incurable disease if the patient or his or her family request it. 30% of the respondents agreed with the statement that people who are dying and who are experiencing extreme suffering should be granted their wish to die whereby a physician gives them a substance which will cause their death.

Only 18.6% of respondents agree with the statement that dying persons who are suffering severely should be granted their wish to die and be enabled to end their own lives. However, 40.1% agree with the statement that physicians should be permitted by law to help a patient who is suffering from an incurable illness and is living with severe pain to end their own life, if the patient asks for it.

### Patients’ rights and physicians’ obligations

51.6% of the respondents agreed with the statement that a dying patient has the right to decide about the end of their life. Moreover, 35.4% of the respondents agreed and 38.5 disagreed with the statement that no one, not even the individual himself/herself, has the right to decide about the moment of his/her death. Furthermore, 39.1% of the respondents agreed and 28.5% disagreed with the statement that physicians are obliged to help a dying patient to realize their wish to die.

### Predictors of the attitudes towards end-of-life practices

A factor analysis, using a component model, varimax rotation and GK dimensionality reduction criterion on 11 statements, that thematically cover end-of life decision-making, identified two factors (patterns of thinking about end-of-life practices). These two factors explain 72.53% of the variance. The first factor we called ‘death self-determination’, and it comprises five items referring to the patients’ right to self-determination regarding death, and the physicians’ corresponding active duties. The second factor we called ‘withholding and withdrawing life-prolonging treatment (WWT) without a patient’s consent’, and it comprises two items related to WWT where the patient’s request dimension is not mentioned (items 1 and 3 in Table 4) in comparison to two similar items (items 2 and 4), where the patient’s request dimension is included. The results of factor analysis are shown in Table 5.

**Table 5:** Matrix of varimax factor structures*

	Death-self-determination	WWT without patient's consent
A dying patient has the right to decide about the end of their own life	.861	
Physicians are obliged to help a dying patient to realize their wish to die	.838	
No one, not even the individual in question, has the right to decide about the moment of their death	− .816	
Procedures in which a person is directly killed by a physician should be absolutely prohibited by law	− .745	
People who are dying and who are experiencing extreme suffering should be granted their wish to die by a physician giving them a substance which will cause their death	.738	
Treatment procedures should not be initiated for people who are dying and who are experiencing extreme and unbearable suffering		.901
All treatment measures should be withdrawn from dying patients who are experiencing extreme and unbearable suffering		.875

*In the first step of factor analysis we excluded the item: “Withholding treatment and "allowing" a patient to die should be regulated by law to avoid abuse”. In the second step of factor analysis we excluded the item: “People who are dying and who are experiencing extreme and unbearable suffering should be granted their wish to die, and all treatment measures that could prolong their life withdrawn.” and the item “People who are dying and who are experiencing extreme and unbearable suffering should have their wish to die granted, and no treatment procedures should be initiated that could extend their life.” In the third step of factor analysis we excluded the item: “People who are dying and who are experiencing extreme suffering should be granted their wish to die, and they should be enabled to end their own lives,” to meet the requirements of a simple structure. The two items: “Do you believe that physicians should be permitted by law to painlessly end the life of a patient who is suffering from an incurable illness, if the patient and the patient’s family ask for it?” and “Do you believe that physicians should be permitted by law to help a patient who is suffering from an incurable illness and is living with severe pain to end their own life, if the patient asks for it?” were not included in the factor analysis because of the different construction of the instrument

We analysed obtained factors (patterns of thinking that emerged) in relation to the basic sociodemographic characteristics of the respondents, in order to determine which respondents are more inclined to which pattern of thinking about end-of-life practices (Table 6). The results show that younger and middle-aged respondents (up to 65 years), respondents with secondary or higher education, those who live in regional centres or in Zagreb, and those with a monthly income of 5.500 to 22.000 HRK view end-of-life decisions through the lens of self-determination. Male respondents and respondents aged 48–64 years were more likely to accept “WWT without a patient’s consent”.

**Table 6:** Results of the analysis of obtained factors (patterns of thinking) in relation to the basic sociodemographic characteristics of the respondents (sex, age, education, monthly income and size of place of residence)

Factors	Sex		Age					Education			Income, HRK (Kuna) per month				Size of place of residence				
	Male	Female	65	64–48	47–31	30		Elementary	High	College and higher	To 5.512.50	5.512.50–11.025.00	11.025.,00–22.050.00	22.050,00 and more	< 2.000	2,001–10,000	10,001–50,000	50,001–500,000	500,001 <
1. «Death self-determination»																			
M	.02849	− .02586	− .58698	.08892	.15559	.25922		− .36073	.08739	.30914	− .27747	.12245	.19706	.22764	− .14398	.01918	− .37831	.33317	.43176
F		0.886			42.480				36.245				16.285				21.950		
p		0.347			< 0.001				< 0.001				< 0.001				< 0.001		
Contrast			65+ < all others					Elementary < all others			1 < 2.3				50,001 and more > all others				
2. «WWT without patient's consent»																			
M	.09389	− .08520	− .12499	.13654	.00371		− .07527	− .03518	.04934	− .10070	− .02743	− .02306	.08488	.03110	.07692	− .04711	− .08385	− .02926	− .08265
F		9.686			4.082				2.059				0.705				1.453		
p		< 0.002			< 0.001				0.128				0.549				0.214		
Contrast	M > F		65+ < 64–48					Elementary > college and higher											

For items number 8 and 10, which were not included in the factor analysis, we used the Chi-square test for analysis to look for possible differences in respondents’ answers in relation to to the basic sociodemographic characteristics of the respondents (Table 7). The results of the analysis of the answers to these two items (Table 7) with regard to the basic sociodemographic characteristics of the respondents (gender, age, level of education, monthly income and size of residence) show that younger respondents (up to 30 years), those with higher education, those who have higher monthly incomes, and those respondents who live in regional centres are more likely to give a positive answer to the item number 8: “*Do you believe that physicians should be permitted by law to painlessly end the life of a patient who is suffering from an incurable illness, if the patient and the patient’s family ask for it?*” Women, older respondents (65 and older), respondents with a lower level of education, those with low monthly income, and those from places with up to 2000 inhabitants, and respondents from towns with up to 50,000 inhabitants are more likely to give a negative answer to this question.

**Table 7:** Differences in respondents’ answers to questions about legal regulation of euthanasia and physician assisted suicide (items 8 and 10) in relation to the basic sociodemographic characteristics of the respondents (sex, age, education, monthly income and size of place of residence) (chi-square test results)

Questions	Sex		Age				Education			Income, HRK (Kuna) per month				Size of Place of Residence				
%	Male	Female	65	64–48	47–31	30	Elementary	High	College and higher	To 5.512,50	5.512,51–11.025,00	11.025,01–22.050,00	22.050,01 and more	< 2.000	2,001–10,000	10,001–50,000	50,001–500,000	500,001 <
1. Question																		
No	30.8	37.5	54.0	31.1	27.9	28.4	39.8	34.1	27.0	42.6	33.2	28.4	25.9	38.2	29.7	50.4	22.9	26.6
Don't know	19.6	15.8	9.3	19.7	24.5	13.2	16.2	19.1	15.2	17.7	18.3	14.9	0	16.0	25.4	15.6	18.4	14.6
Yes	49.5	46.7	36.7	49.1	47.6	58.4	43.9	46.8	57.8	39.7	48.5	56.8	74.1	45.7	44.9	34.1	58.7	58.9
Chi-square	χ^2^ = 6.591; df = 2; p < 0.04		χ^2^ = 68.148; df = 6; p < 0.001				χ^2^ = 13.207; df = 4; p < 0.01			χ^2^ = 29.167; df = 6; p < 0.001				χ^2^ = 45.833; df = 8; p < 0.001				
2. Question																		
No	32.8	41.6	53.8	33.4	31.1	35.7	44.6	36.3	29.6	43.5	36.9	30.3	37.0	41.3	32.2	53.4	27.5	28.8
Don't know	22.0	19.4	15.3	24.8	23.1	16.8	21.3	20.7	19.2	19.8	21.4	18.1	18.5	22.4	22.4	15.0	24.7	12.8
Yes	45.2	39.0	30.9	41.8	45.8	47.5	34.1	43.0	51.2	36.8	41.7	51.6	44.4	36.3	45.4	31.6	47.8	58.3
Chi-square	χ^2^ = 9.338; df = 2; p < 0.009		χ^2^ = 40.829; df = 6; p < 0.001				χ^2^ = 17.098; df = 4; p < 0.002			χ^2^ = 14.992; df = 6; p < 0.02				χ^2^ = 48.385; df = 8; p < 0.001				

Furthermore, men, respondents under the age of 47, respondents with a higher level of education, those with a high monthly income, and those from regional centres are more likely to give an affirmative answer item number 10: “*Do you believe that physicians should be permitted by law to help a patient who is suffering from an incurable illness and is living with severe pain to end their own life, if the patient asks for it?*” Women, older respondents, those with a lower level of education, those with low monthly incomes, and respondents from smaller towns (up to 2,000 inhabitants), as well as those from cities up to 50,000 inhabitants are more likely to give a negative answer to this question.

Table 8 shows the results of multiple regression analysis, where individual factors were analysed with respect to one predictor set covering different areas. The results show that the selected predictor set interprets about 55% of the variance of the first factor ("death self-determination"), and a statistically significant correlation was obtained with 12 separate predictors. Respondents who approve of homosexuality, those who do not attend religious ceremonies, those who do not see the meaning of life in believing in God, those who do not believe in hell, and those who do not contribute to the community are more likely to view end-of-life decisions through the lens of self-determination. Furthermore, the following categories of respondents are also likely to view end-of-life decisions through the lens of self-determination: those who are of "liberal" political orientation, those who approve of abortion, who think that solving unfinished business and avoiding being a burden to one’s family and loved ones are important characteristics of a good death, those who have not experienced the death of their own child, those who have had the experience of death of a pet, and respondents who do not think there is trust and mutual respect between physicians and patients.

**Table 8:** Results of multiple regression analysis- predictors of view on end-of-life decisions through the lens of self-determinataion and of accpetance of WWT without patient’s' consent*

Factors	R^2^	Predictors	*Standardized coefficients*	*p*
			*beta*	
1. «Death self-determination»	0.546	Approval of homosexuality	0.205	< 0.001
		Church Attendance	− 0.232	< 0.001
		Death can have meaning only when a person believes in God	− 0.169	< 0.001
		Belief in hell	− 0.225	< 0.002
		The meaning of life is to contribute to the life of the community	− 0.161	< 0.001
		Worldview—"liberal" vs "conservative"	− 0.157	< 0.001
		Approval of abortion	0.100	< 0.010
		Characteristic of a good death—to solve all unfinished business / things	0.087	< 0.004
		Characteristic of a good death—not to be a burden to family / relatives	0.070	< 0.025
		Death of a pet	0.085	< 0.004
		Death of one's own child	− 0.089	< 0.004
		There is trust and mutual respect between physicians and patients in Croatia	− 0.077	< 0.040
2. «WWT without patient's consent»	0.202	Approval of casual sex	0.226	< 0.001
		The characteristic of a good death is to be at peace with God	− 0.176	< 0.001
		Death is uncertain and unknown, it is pointless to think about it at all	0.140	< 0.002
		The state of health of the respondent	0.125	< 0.004
		The meaning of life is in the self-realization of one's own possibilities	0.132	< 0.002
		Death of a loved one	− 0.118	< 0.007

*The predictor set covers a wide range of topics, from the meaning of life and death, the characteristics of good death and the experience of death and dying, subjective assessment of one's health, through general trust, trust in the health system and physicians, to religiosity in general, political orientations and tolerance of personal choice. The predictor circuit contains a total of 55 items

The selected predictor set interprets about 20% of the variance of the second factor (“WWT without a patient’s consent”), and statistical significance was obtained for 6 separate predictors. The results show that respondents who approve of casual sex, those who do not consider reconciliation with God an important characteristic of a good death, those who state that they are in good health, and those who have not experienced the death of a loved one, are more likely to accept “WWT without the patient’s consent”. Respondents who believe that death is unknown and uncertain, and that it is completely meaningless to think about death and who see the meaning of life in the self-realization of their own possibilities, are also more inclined to accept “WWT without the patient’s consent”.

Finally, the results of the correlation analysis (bivariate correlation, Pearson's coefficient) (Table 9) clearly show that respondents who agree that physicians should be allowed by law to painlessly end the life of a patient suffering from an incurable disease, as required by the patient and their family, as well as those who agree that physicians should be allowed by law to help a patient suffering from an incurable disease and living in severe pain to end their own life at their personal request, are more likely to view end-of-life decisions through the lens self-determination and little bit less likely to accept WWT without the patient’s consent.

**Table 9:** Correlation between items 8 and 10 and two factors “death-self-determination” and “WWT without a patient’s consent”

	Death-self-determination	WWT without patient's consent
Item 8: Do you believe that physicians should be permitted by law to painlessly end the life of a patient who is suffering from an incurable illness, if the patient and the patient’s family ask for it?	0.699**	0.293**
Item10: Do you believe that physicians should be permitted by law to help a patient who is suffering from an incurable illness and is living with severe pain to end their own life, if the patient asks for it?	0.708**	0.254**

**p < 0.001

## Discussion

This is the first study on a representative sample of the Croatian population with regards to attitudes on withholding /withdrawing life-prolonging treatment, euthanasia, assisted suicide and physician assisted suicide. In addition, the study analyses the connection between these attitudes and socio-demographic characteristics, religion, political orientation, tolerance towards freedom and personal choice, trust in physicians, health status, experiences with death and caring for seriously ill patients, and attitudes towards death and dying.

### Withdrawing and withholding life-prolonging treatment

Only 19.8% of the Croatian general public agree with withholding treatment procedures in general and 14.4% with withdrawing of all treatment procedures from dying people who are experiencing extreme and unbearable suffering.

When it comes to withholding life-prolonging treatment, 38.1% of the Croatian general public approve withholding such treatment in order to grant the wishes of dying people who are experiencing extreme and unbearable suffering. In comparison, 66.5% of the general public in Korea supported this option [27]. However, the Korean study made specific distinctions between different end-of-life interventions, such as active pain control, withdraw of futile life sustaining treatment, and withholding of life-sustaining measures, which we did not. Thus interpretation of our respondents’ answers leaves a certain amount of ambiguity.

Only 37.8% of the Croatian general public agree with respecting the wishes of dying people who are experiencing extreme and unbearable suffering and withdrawing life-prolonging treatment in comparison to 88.7% of the Korean general public who would support this procedure [27]. In a similar Austrian study, 77.4% of the general public approved withholding or withdrawing life-prolonging treatment upon the patient’s autonomous request. [28]. Also, in the study by Steinberg et al., in Australia 78% of the general public supported withdrawing life-prolonging treatment on the basis of the patients’ wishes [29]. The same was observed in a Canadian study by Singer et al., where the general public expressed high rates of approval of withdrawing life-prolonging treatment for a patient unlikely to recover (85% for a competent patient, 88% for an incompetent patient, who had expressed his/her wishes in advance through a living will, and 76% for an incompetent patient based on the family's request) [30]. In a Canadian study by Marcaux et al., 85.8% of the general population in Quebec supported treatment withdrawal upon the patient’s request [31] and in a German study, 78% of the population approved withholding or withdrawing treatment upon the patient’s request [32]. In the Hong Kong study by Study Chong et al. the general population had a neutral position when it comes to withholding or withdrawing life-prolonging treatment—they did not approve or disapprove of it [24]. In a Swedish study, only 40.2% of the general public would withhold life-prolonging treatment, and 77% were prepared to withdraw life-prolonging treatment to an incompetent patient [33]. The Croatian general public is less likely to accept WWT in comparison with other countries.

It seems that half of the Croatian general public would support a dying person’s autonomous wishes when it comes to end-of-life practices. However, middle-aged respondents and male respondents displayed a certain level of paternalism, and were more likely to accept WWT without the patient’s consent. That is why our factor analysis identified a factor dealing with withholding and withdrawing life-prolonging treatment without the patient’s consent. Moreover, the Austrian and Swedish studies did not find any gender differences, as our study did when it comes to acceptance of WWT [28, 33]. The respondents’ opinions in our study were also divided as to whether physicians should be involved in end-of-life decision-making, and whether they are obliged to help a dying patient to realize their wish to die.

Just as in the Korean study, we found that persons who found the meaning of life in the self-realization of their own possibilities are likely to accept withholding/ withdrawing of life-prolonging treatment [26]. In our study, the respondents were also in favour of withholding and withdrawing treatment who thought that death is uncertain and unknown, that it is pointless to think about it at all, and those who were in good health. Those who had experienced the death of a loved one and think that a good death means being at peace with God were against withholding/withdrawing treatment. Previous studies have shown that personal experience regarding caring for a seriously ill or dying person, as well as religious views can influence a person’s acceptance or rejection of WWT [28, 33–35].

Our respondents expressed the fear of abuse when it comes to the practice of withholding life-prolonging treatment, and therefore opted for its regulation by law. This is not unexpected since withholding/ withdrawing life-prolonging treatment may raise many questions for patients, families and physicians: the patient’s decisional capacity, the patient’s true wishes when it comes to end-of-life procedures, family involvement, the role of the physician in the process of decision-making, and physician–patient and family communication [36, 37].

### Euthanasia, assisted suicide and physician-assisted suicide

The attitudes of the Croatian population towards euthanasia have been tracked through the European Values Study project [17]. The acceptance of euthanasia increased from 3.82 in 1999, to 4.00 in 2008, and to 4.37 in 2017 (on a Likert scale of 1 to 10). The percentage of the population that believes that euthanasia cannot be justifiable under any circumstances (50.9% in 1999, 44.4% in 2008 and 42.7% in 2017) is still very high [38, 39]. Our research showed a small drop in agreement with the absolute prohibition of the practice by law (37.2% of the respondents). However, our questions were framed in a different way, not explicitly mentioning the term euthanasia, but rather describing it (items 6, 7, 8 in Table 4), as suggested in the literature [27]. One of the items describing the practice of euthanasia was also framed slightly ambiguously: “*Do you believe that physicians should be permitted by law to painlessly end the life of a patient who is suffering from an incurable illness, if the patient and the patient’s family ask for it?*” thus combining the practices of euthanasia requested by patient and the practice ending of life without explicit patient consent. We found a higher level of agreement in the general public than for the items describing the practice of euthanasia requested by the patient alone. Moreover, the description of the practice in this item was different than in the other two items. Here we described euthanasia as: “*a physician painlessly ending the life of a patient*” in contrast to “a *physician killing a person”* and “a *physician giving a substance which will cause the death of the person*”.

Respondents who hold a more open approach to euthanasia tend to endorse a liberal worldview, be less religious, have a higher level of education, be younger, and be from regional centres, which is in accordance with similar studies in Europe [7, 10, 27], the USA [10] and Canada [10, 30, 31], and in the studies done in South Korea [26] and Hong Kong [24]. Not being a burden to the family was seen an important trait of a good death for those in favour of euthanasia in our study, as in some other studies [26, 34]. In our study, respondents in favour of euthanasia did not have any experience of the death of a loved one or caring for a seriously ill person. However, the available evidence regarding the associations of such experiences with views on euthanasia or assisted suicide is rather mixed, with some studies finding positive, no or negative [40] associations between personal experiences with the terminal illness of a relative or friend and/or being a family caregiver on the one hand and attitudes towards euthanasia or assisted suicide on the other. [5, 25, 27, 34, 40–42]. Moreover, respondents in favour of euthanasia did not find that there is trust and mutual respect between physicians and patients in Croatia, and see their self-determination in end-of-life practice as a form of control. This is in contrast with research done by Köneke, where the level of trust in the healthcare system was strongly positively linked to attitudes towards euthanasia [43]. Similarly, in the Swedish study of Lindblad et al. the general population believed that their trust in the medical services would increase (38%) or not be influenced at all (45%) if physician-assisted suicide were to be allowed. However, 75% of those who were against physician-assisted suicide believed that their trust would decrease [44].

In our study, assisted suicide was not seen as an acceptable end-of-life procedure while physician assisted-suicide had more proponents (more than double in comparison to assisted suicide) in the general public. On the other hand, Parpa et al. found in their study among Greek health care professionals and the relatives of advanced cancer patients, that they opted more for a tailored, professional care approach, rending physician-assisted suicide unnecessary in their case [45]. In the research done by Kouwenhoven et al. and Hendin, physicians and patients preferred euthanasia to physician-assisted suicide as a safer and more certain way of terminating life [46, 47].

### Limitations

In our study we used quantitative methods which provided us with an overview of the general public’s attitudes on withholding and withdrawing life-prolonging treatment, euthanasia and assisted suicide. This methodology did not give us any in-depth insight into the argumentations on which these attitudes were founded. Therefore, more qualitative research on the general population’s attitudes towards end-of-life decisions is recommended.

In addition, we only investigated the general public’s views on withholding and withdrawing life-prolonging treatment, euthanasia, assisted suicide and physician assisted suicide. Additional research is needed, using both quantitative and qualitative methodologies, on the attitudes towards the end-of-life practices of more specific groups, such as healthcare professionals, the families of seriously ill and dying patients, and the patients themselves, for a better understanding of practices, and specific problems and concerns.

Finally, some of our wording was not clear enough and items relating to withholding or withdrawing treatment, but also euthanasia, were possibly ambiguous, giving rise to a clear caveat when it comes to interpreting items number 3 and 8. In the item 3 the fact that pain and symptom management would continue after other forms of treatment are withdrawn from dying patients who are experiencing extreme and unbearable suffering was not clearly stated. In the item 8 distinctions should have been made between the possible legalisation of the practices of euthanasia requested by patient and the practice ending of life without explicit patient consent, thus dividing item 8 in two separate items.

## Data Availability

The datasets generated and/or analyzed during the current study are not publicly available due to limitations of ethical approval involving the participants’ data and anonymity but are available from the corresponding author on reasonable request.
